# Lack of an Efficient Endoplasmic Reticulum-localized Recycling System Protects Peroxiredoxin IV from Hyperoxidation*

**DOI:** 10.1074/jbc.M113.529305

**Published:** 2014-01-08

**Authors:** Zhenbo Cao, Suraj Subramaniam, Neil J. Bulleid

**Affiliations:** From the Institute of Molecular, Cell, and Systems Biology, College of Medical, Veterinary and Life Science, University of Glasgow, Glasgow G12 8QQ, United Kingdom

**Keywords:** Disulfide, Peroxiredoxin, Protein Isomerase, Thioredoxin, Thioredoxin Reductase

## Abstract

Typical 2-Cys peroxiredoxins are required to remove hydrogen peroxide from several different cellular compartments. Their activity can be regulated by hyperoxidation and consequent inactivation of the active-site peroxidatic cysteine. Here we developed a simple assay to quantify the hyperoxidation of peroxiredoxins. Hyperoxidation of peroxiredoxins can only occur efficiently in the presence of a recycling system, usually involving thioredoxin and thioredoxin reductase. We demonstrate that there is a marked difference in the sensitivity of the endoplasmic reticulum-localized peroxiredoxin to hyperoxidation compared with either the cytosolic or mitochondrial enzymes. Each enzyme is equally sensitive to hyperoxidation in the presence of a robust recycling system. Our results demonstrate that peroxiredoxin IV recycling in the endoplasmic reticulum is much less efficient than in the cytosol or mitochondria, leading to the protection of peroxiredoxin IV from hyperoxidation.

## Teaser

**Background:** Peroxiredoxin IV metabolizes endoplasmic reticulum-derived hydrogen peroxide.

**Results:** In contrast to other peroxiredoxins, peroxiredoxin IV is insensitive to hyperoxidation within its cellular environment.

**Conclusion:** There is a lack of a robust peroxiredoxin IV recycling system within the endoplasmic reticulum.

**Significance:** Studying the reduction of peroxiredoxin IV highlights the different mechanisms for maintaining redox status within different cellular compartments.

## Introduction

**FIGURE 1.**
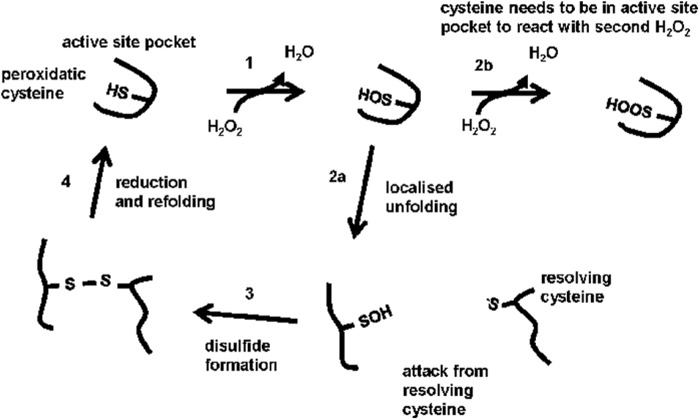
**Reaction cycle and hyperoxidation of the 2-Cys Prxs.** Schematic depicting the reaction cycle of the 2-Cys family of Prxs. *1*, the peroxidatic cysteine reacts with a H_2_O_2_ molecule when present in the fully folded active site pocket to become sulfenylated. *2a*, the active site unfolds, thereby preventing further modification of the peroxidatic cysteine. *2b*, a second H_2_O_2_ molecule can react with the peroxidatic cysteine but only if it reacts before the active site pocket unfolds. *3*, the resolving cysteine reacts with the sulfenylated peroxidatic cysteine to form a disulfide. *4*, the disulfide is reduced, and the active site pocket refolds. The hyperoxidation of the Prxs is dependent upon the rapid reformation of the active site pocket, which requires efficient reduction of the disulfide.

Peroxiredoxin (Prxs)^2^ are a family of ubiquitous, highly expressed antioxidant enzymes capable of efficiently metabolizing hydrogen peroxide (H_2_O_2_) (1). They are the dominant cellular peroxide-reducing enzymes on the basis of their relative reactivity and protein concentration (2). In human cells, there are six Prxs that differ in subcellular localization and number of catalytic cysteine residues (3). The typical 2-Cys Prxs are localized to the cytosol (PrxI and II), the mitochondrion (PrxIII), and the endoplasmic reticulum (ER) (PrxIV). Under normal conditions, the active-site peroxidatic cysteine rapidly cycles between a sulfenic acid, disulfide, and free thiol, with the reduction of the disulfide being catalyzed by a member of the thioredoxin family of enzymes (3) (Fig. 1). The reactivity of the peroxidatic cysteine toward H_2_O_2_ is dependent upon its presence in the folded active site (4). Upon sulfenylation, there is a local unfolding of the active site to allow the peroxidatic cysteine to form a disulfide with a resolving cysteine located outside of the active site. Crucially, this localized unfolding reduces the reactivity of the peroxidatic cysteine because it is no longer in the optimal environment for reaction with H_2_O_2_. When the disulfide has been reduced, the active site refolds to allow reaction with the next H_2_O_2_ molecule. In the presence of high concentrations of H_2_O_2_, the peroxidatic cysteine can become hyperoxidized to form cysteine sulfinic acid, a modification that renders the protein inactive. Hyperoxidation occurs in competition with disulfide formation and can, therefore, occur more efficiently at high H_2_O_2-_ concentrations. Although recycling is not essential for hyperoxidation (5), at lower H_2_O_2_ concentrations, disulfide formation occurs more efficiently, requiring recycling to allow hyperoxidation to occur (6, 7). This recycling is usually catalyzed by a thioredoxin domain-containing protein. It has also been shown that the hyperoxidized, sulfinylated active-site cysteine can be recycled back to the thiol and, thereby, reactivated by sulfiredoxin, an ATP-dependent enzyme localized to the cytosol (8). The inactivation of Prxs by hyperoxidation of the active-site cysteine residue has led to the suggestion that this modification provides a mechanism to allow H_2_O_2_ to act as a signaling molecule (3).

PrxIV reduces H_2_O_2_ produced by the ER oxidase 1 (Ero1) (9). The oxidized PrxIV formed can pass the disulfide bond to a member of the protein disulfide isomerase (PDI) family (10, 11). Therefore, during reduction of one molecule of oxygen to two molecules of water, two disulfide bonds can be introduced to PDI family members by Ero1 and PrxIV. The crystal structure of PrxIV shows that it forms a stable decamer and shares the same active sites as PrxI-III (4). Also like other Prxs, the reactivity of PrxIV toward H_2_O_2_ is high (12), making PrxIV the likely primary target of H_2_O_2_. Under conditions where Ero1 is activated, such as following treatment of cells with DTT, PrxIV can become hyperoxidized (9), although the physiological relevance of this modification is uncertain. In addition, it is unknown whether the ER contains a sulfiredoxin capable of recycling any sulfinylated PrxIV.

We developed a simple assay on the basis of one-dimensional isoelectric focusing (IEF) that allows us to monitor the hyperoxidation of all intracellular Prxs simultaneously. The ability to quantify the oxidation status of the Prxs enabled us to monitor the sensitivity of the Prxs to hyperoxidation within distinct cellular compartments. Here we show that ER-localized PrxIV is insensitive to hyperoxidation even at concentrations of H_2_O_2_ that cause hyperoxidation of Prxs localized to the cytosol and mitochondria. In addition, we could not find any evidence for the existence of an ER-localized sulfiredoxin.

## EXPERIMENTAL PROCEDURES

### 

#### 

##### Reagents and Antibodies

An antibody was raised in rabbits to a peptide with the sequence of the first 11 N-terminal amino acids of mature PrxIV (WETEERPRTRC). The commercial rabbit polyclonal antibodies used were anti-Prx II and III (Abcam) and anti-Prx-SO_2–3_ (AbFrontier). An anti-PrxIV mouse monoclonal antibody (Abcam) was also used. The fluorescently conjugated anti-mouse and anti-rabbit IgG 680 and 800 secondary antibodies were purchased from either Li-Cor IRDye or Thermo-Scientific DyeLight. Human recombinant PrxIV, human PDI, *Escherichia coli* thioredoxin, and *Saccharomyces cerevisiae* thioredoxin reductase were purified as described previously (4, 9, 10, 13).

##### Preparation of Samples for IEF

Cells were maintained in DMEM supplemented with 10% fetal bovine serum before being treated with 20 mm *N*-ethylmaleimide to prevent disulfide exchange. Cells were lysed in buffer A (50 mm Tris-HCl buffer (pH 8.0) containing 150 mm NaCl, 5 mm EDTA, and 1% (w/v) Triton X-100). Cell lysates were centrifuged (10,000 × *g* for 10 min at 4 °C) to remove insoluble material, and 5 volumes of ice-cold acetone were added to the supernatant and incubated for at least 2 h at −20 °C. The protein precipitated was isolated by centrifugation (10,000 × *g* for 10 min at 4 °C) and washed with 80% ice-cold acetone. The precipitate was isolated by centrifugation, and the pellet was air-dried and suspended in IEF sample buffer consisting of 7 m urea, 2 m thiourea, 2% (w/v) CHAPS, 0.8% (v/v) ampholytes (pH 4–6), 50 mm DTT, 4% (v/v) glycerol, and 0.02% (w/v) bromphenol blue. The protein was dissolved in IEF sample buffer for 1 h at room temperature before application to a one-dimensional IEF gel.

##### One-dimensional IEF

A 10% gel containing 9 m urea, 0.4% (v/v) ampholytes mix (pH4–6), and 1% (w/v) CHAPS was cast and run using the mini-VE system (Amersham Biosciences). The cathodic buffer was 10 mm phosphoric acid, and the anodic buffer was 25 mm Tris base. One-dimensional IEF was carried out at 1000 V and 2 mA for 4 h. Focused gels were rinsed in water and soaked in transfer buffer for 20 min at room temperature.

##### Immunoblotting

Following one-dimensional IEF, proteins were transferred to a nitrocellulose membrane (Li-Cor Biosciences). Nonspecific binding was blocked using 5% (w/v) nonfat dried skimmed milk in Tween/Tris-buffered saline (TTBS) (50 mm Tris buffer (pH 7.5) containing 150 mm NaCl and 0.1% (v/v) Tween 20). Membranes were incubated with primary antibody for 16 h at 4 °C in TTBS. The secondary antibody was diluted 1:5000 in TTBS, and incubations were performed in a light-shielded box for 45 min at room temperature. Specific proteins were visualized using an Odyssey SA imaging system (Li-Cor Biosciences). For quantification of fluorescent immunoblot analyses, scans were performed at the minimum intensities required to see all relevant proteins. Densitometry was then performed on unmodified output images using ImageJ (National Institutes of Health) (fraction hyperoxidized = SO_2_H/(SH + SO_2_H)). Statistical analysis was carried out using a one-tailed, paired *t* test assuming unequal variance between samples.

##### Metabolic Labeling and Pulse-Chase Analysis

Cells were starved for 30 min in cysteine/methionine-free DMEM and then radiolabeled in the same medium containing 22 μCi/ml EXPRESS^35^S protein labeling mix (PerkinElmer Life Sciences). After 30 min of incubation at 37 °C, the radiolabel was removed, and cells were washed with PBS and incubated in complete DMEM for various lengths of time. Cells were washed with PBS before being lysed in buffer A. Cell debris was removed by centrifugation (10,000 × *g* for 10 min at 4 °C). The lysates were precleared by adding protein A-Sepharose (Generon) and incubated for 30 min at 4 °C. For immunoisolation, anti-PrxIV antibody was added to protein A-Sepharose. Samples were incubated for 16 h at 4 °C with end-over-end mixing. The Sepharose beads were pelleted by centrifugation (600 × *g* for 1 min) and washed three times with buffer A. Proteins were eluted with IEF sample buffer prior to loading onto a one-dimensional IEF gel. Gels were fixed, dried, and exposed to a phosphorimaging plate. Radioactivity was detected using a Fujifilm FLA-7000 PhosphorImager. Quantification of band intensities was carried out using ImageJ software.

##### In Vitro Assays

Recombinant human PrxIV (2.8 μm) was expressed and purified as described previously (9) and mixed with GSH (1 mm), GSH/GSSG (3:1,1 mm), reduced PDI (2.8 μm), DTT (1 mm), or a thioredoxin recycling system (2.8 μm thioredoxin, 2.8 μm thioredoxin reductase, and 1.5 mm NADPH) in 50 mm Tris buffer (pH 7.5) containing 150 mm NaCl and 1 mm EDTA. Various concentrations of H_2_O_2_ were added, and samples were incubated for 10 min at 30 °C. IEF sample buffer (5 volumes) was added to each sample and mixed for 1 h before being loaded onto a one-dimensional IEF gel. An immunoblot analysis using the polyclonal PrxIV antibody was carried out, and band intensities were quantified using ImageJ software.

## RESULTS

### 

#### 

##### Quantitative Assay for the Hyperoxidation of Prxs

**FIGURE 2.**
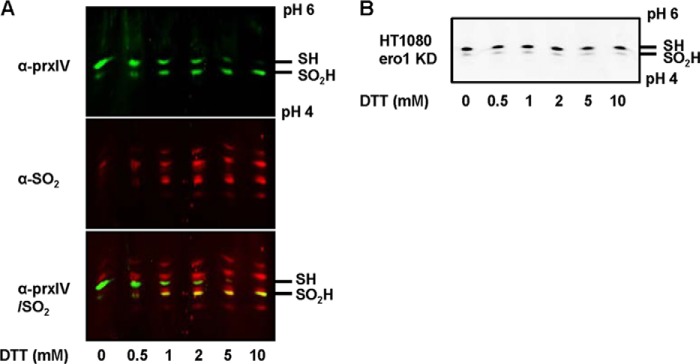
**Assay for Prx hyperoxidation in HT1080 and Ero1-depleted cells.** HT1080 cells (*A*) or Ero1-depleted cells (*B*) were treated with varying concentrations of DTT, as indicated, for 5 min at 37 °C. Cells were lysed, and samples were resolved on a one-dimensional IEF gel prepared using pH 4–6 ampholytes. After one-dimensional IEF, samples were transferred to nitrocellulose, and immunoblots were probed with either a mouse monoclonal (*A*, *top panel*, α*-prxIV*), a rabbit polyclonal antibody to Prx IV (*B*), or a rabbit polyclonal antibody to sulfinylated Prxs (*A*, *center panel*, α*-SO_2_*). In *A*, blots were developed with a green fluorescent anti-mouse IgG antibody (*top panel*) or a red fluorescent anti-rabbit IgG antibody (*center panel*). Images are from the same blot, with the *bottom panel* being an overlay of the *top* and *center panels*, illustrating overlap (*yellow*). The blot in *B* was developed with an anti-rabbit secondary antibody. The acidic, hyperoxidized form of PrxIV (*SO_2_H*) is resolved from the unmodified more basic form (*SH*). Each experiment was carried out three times with similar results.

The hyperoxidation of the members of the typical 2-Cys family of Prxs has been detected previously, either by using an antibody (α-SO_2_) that recognizes the sulfinylated active-site peptide of all 2-Cys Prxs (14) or by separation of the various oxidation states of the proteins by two-dimensional gel analysis (15). Although the antibody approach is very sensitive and provides a qualitative analysis of hyperoxidation, it suffers from a lack of specificity toward individual members of the family (9) and is non-quantitative in that it cannot assess the fraction of the total peroxiredoxin hyperoxidized. Such quantification can be provided by a two-dimensional gel analysis, but this approach is not suitable for screening large numbers of samples (16). To overcome this problem, we used a one-dimensional IEF approach that separates the unmodified or sulfenylated forms from the sulfinylated proteins on the basis of charge. To validate this approach, we treated HT1080 cells with increasing concentrations of DTT, separated the resulting cell lysates by one-dimensional IEF, and visualized specific proteins with either an antibody to PrxIV (α-PrxIV) or the α-SO_2_ antibody (Fig. 2*A*). We have shown previously that DTT generates H_2_O_2_ in the ER by an Ero1-dependent mechanism and leads to hyperoxidation of PrxIV (9). Because the antibodies are raised in different species, we could use different fluorescently labeled secondary antibodies on the same blot to determine the comigration of proteins. As the concentration of DTT increases, so does the appearance of a more acidic form of PrxIV (Fig. 2*A*, *top panel*) that comigrates with protein recognized by the α-SO_2_ antibody (*bottom panel*). The additional bands seen with the α-SO_2_ antibody (*center panel*) reflect cross-reactivity of this antibody with other 2-Cys Prxs that show some increased reactivity at high concentrations of DTT (see below). Strikingly, at higher concentrations of DTT, most of the PrxIV was modified. To demonstrate that PrxIV was being modified following DTT treatment by the Ero1-dependent production of H_2_O_2_, we repeated the experiment with a cell line that contains low levels of Ero1α because of stable shRNA knockdown (17). No increase in hyperoxidized PrxIV was seen, even at high concentrations of DTT (Fig. 2*B*). Hence, the one-dimensional IEF system can separate unmodified from sulfinylated PrxIV and can be used to specifically quantify the levels of hyperoxidized PrxIV as well as other members of the 2-Cys Prx family.

##### Recycling of Hyperoxidized PrxIV

**FIGURE 3.**
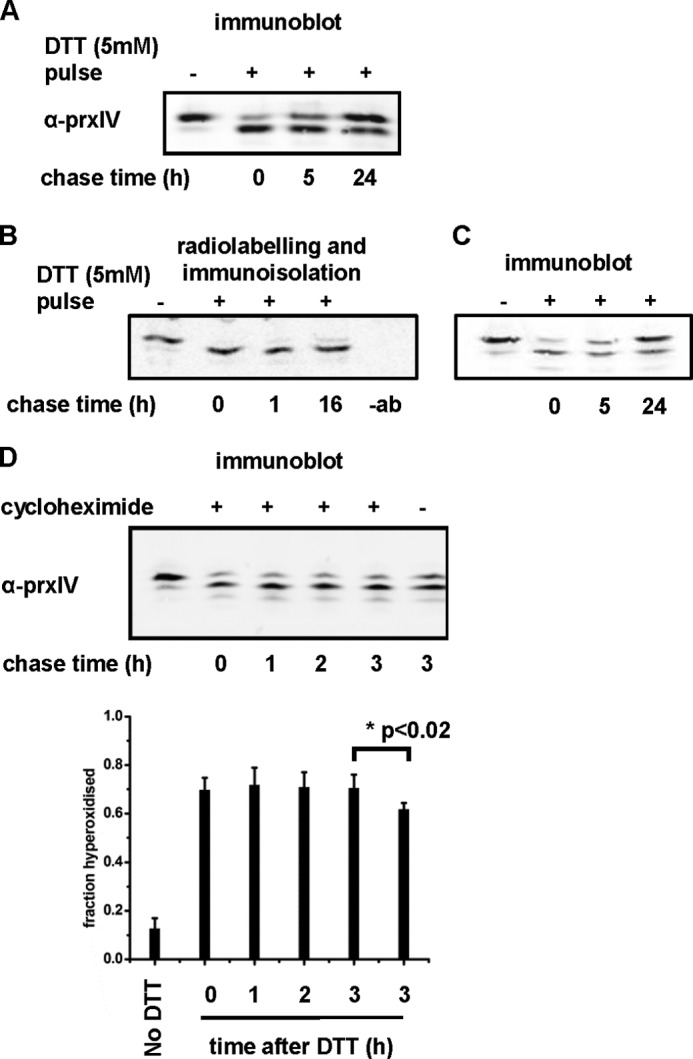
**Recycling of hyperoxidized PrxIV.** HT1080 cells (*A* and *D*) or a stable HT1080 cell line overexpressing PrxIV (*B* and *C*) were treated with DTT (5 mm) for 10 min at 37 °C to induce hyperoxidation of PrxIV. DTT was removed, cells were incubated in complete DMEM for the indicated time, and cell lysates were prepared as described in Fig. 2. *A*, immunoblot analysis of HT1080 indicating some recovery of unmodified PrxIV. *B*, cells overexpressing PrxIV were radiolabeled for 30 min, followed by treatment with DTT (5 mm). PrxIV was immunoisolated by using a polyclonal antibody to PrxIV. A control sample, isolated without antibody, was also processed (-*ab*). The samples were separated on a one-dimensional IEF gel, and the dried gel was exposed to phosphorimaging analysis. *C*, immunoblot analysis of the PrxIV-overexpressing cell line indicating some recovery of unmodified PrxIV. *D*, immunoblot analysis of HT1080 cells either treated with cycloheximide (0.5 mm) or not treated, as indicated. Cells were exposed to cycloheximide after treatment with DTT (5 mm) for the indicated times. The average of the fraction of PrxIV hyperoxidized from three independent experiments was calculated and plotted against time after DTT treatment. *Error bars* indicate the mean ± S.D. The statistical significance of the difference between the 3-h chase time with and without cycloheximide is as indicated.

The modification of the 2-Cys Prxs to the sulfinylated form blocks reactivity of the active-site peroxidatic cysteine (7). However, a cytosolic enzyme called sulfiredoxin has been identified that catalyzes recycling of the sulfinylated peroxidatic cysteine back to an active thiol (8). To investigate whether hyperoxidized PrxIV could also be recycled, we first treated cells with a pulse of DTT to hyperoxidize about 80% of the protein (Fig. 3*A*), removed the DTT, and determined the recovery over 24 h. The hyperoxidized fraction dropped to <40%, indicating that either the pre-existing PrxIV was being recycled or that newly synthesized protein was replacing the hyperoxidized PrxIV. To determine whether pre-existing hyperoxidized PrxIV was being recycled, we first radiolabeled newly synthesized protein for 30 min, treated it with DTT to hyperoxidize the PrxIV, and chased it for up to 16 h in fresh medium. For this experiment, we used a cell line overexpressing PrxIV (18) because the endogenous protein was not radiolabeled efficiently. We immunoisolated the radiolabeled PrxIV at various time points and separated the oxidized forms of PrxIV by one-dimensional IEF (Fig. 3*B*). None of the hyperoxidized PrxIV radiolabeled during the pulse period was recycled, suggesting that, for PrxIV, the only mechanism to regenerate the active enzyme following hyperoxidation is by new protein synthesis. An immunoblot analysis of the recovery of PrxIV from hyperoxidation in this overexpressing cell line gave a similar result as that from HT1080s (Fig. 3*C*). That this recovery was due to new protein synthesis was confirmed when we analyzed the recovery of hyperoxidized PrxIV following a DTT pulse in the presence of the protein synthesis inhibitor cycloheximide (Fig. 3*D*). Some recovery was observed after 3 h in the absence of cycloheximide, but no recovery was seen in the presence of the protein synthesis inhibitor. Taken together, these results strongly suggest that there is no sulfiredoxin activity to recycle hyperoxidized PrxIV in the ER.

##### Differential Hyperoxidation of Prxs Located in Different Intracellular Compartments

**FIGURE 4.**
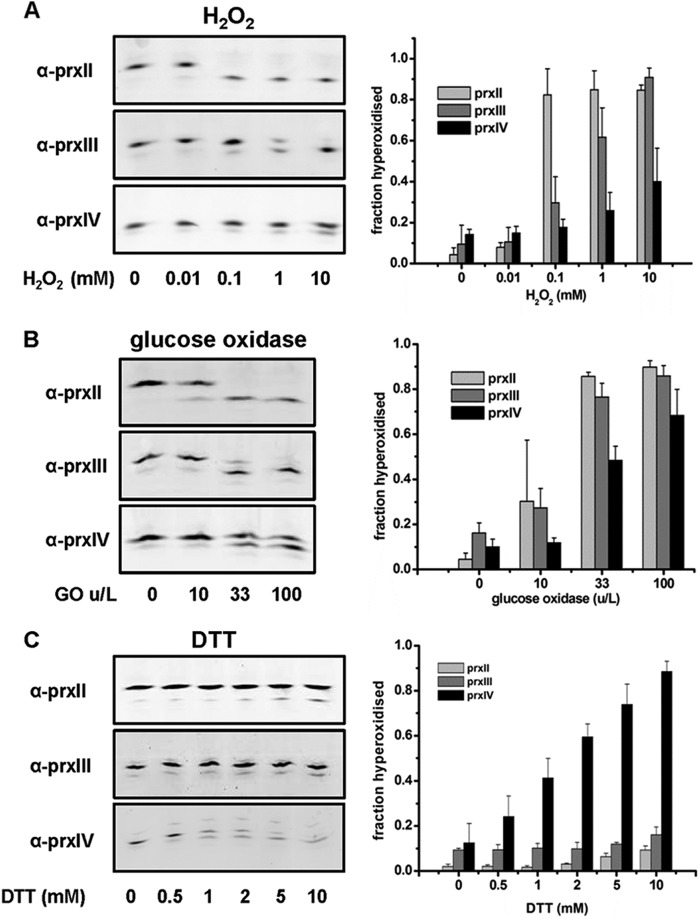
**Differential sensitivity of Prxs located in different intracellular compartments to hyperoxidation.** HT1080 cells were exposed to increasing concentrations of H_2_O_2_ (0–10 mm) for 10 min (*A*), units of glucose oxidase (0–100 units/l) for 3 h (*B*), or concentrations of DTT (*C*). Cell lysates were prepared as described in Fig. 2 and separated by one-dimensional IEF. Immunoblot analyses were developed with polyclonal antibodies to PrxII (α-*prxII*), PrxIII (α-*prxIII*), or PrxIV (α-*prxIV*), as indicated. The fraction of hyperoxidized Prx for the displayed image was calculated and plotted graphically for each treatment. Each experiment was carried out in triplicate with similar results obtained. *Error bars* indicate the mean ± S.D.

The ability to quantify the extent of hyperoxidation of 2-Cys Prxs enabled us to evaluate the consequence of the addition of H_2_O_2_ to HT1080 cells on the hyperoxidation of Prxs localized to the cytosol (PrxII), mitochondria (PrxIII), or the ER (PrxIV). A marked transition from mostly unmodified (basic) to mostly hyperoxidized (acidic) PrxII was seen after the addition of 100 μm H_2_O_2_ (Fig. 4*A*). As demonstrated previously, PrxIII was slightly less sensitive to hyperoxidation, requiring higher concentrations to become hyperoxidized (5, 19). PrxIV was largely resistant to hyperoxidation, with only low levels of hyperoxidation, even at 10 mm H_2_O_2_. The difference in sensitivity to hyperoxidation between PrxII and PrxIV was statistically significant (*p* < 0.01 at 0.1 and 1 mm H_2_O_2_). Similar differential susceptibility of the Prxs to hyperoxidation was also observed when the experiment was repeated with HeLa cells (results not shown). The addition of H_2_O_2_ exogenously to cells is unlikely to reflect a physiological increase in concentration, so to generate a more gradual increase, we added various concentrations of glucose oxidase to the cell medium to generate H_2_O_2_ *in situ* and assayed the extent of hyperoxidation after 3 h (Fig. 4*B*). Similar differential hyperoxidation of the Prxs in different cellular compartments was observed, with PrxIV being less sensitive to hyperoxidation than PrxII or PrxIII (*p* < 0.01 at 33 units/liter and < 0.05 at 100 units/liter, comparing PrxII to PrxIV). Taken together, these results demonstrate that there are distinct sensitivities to hyperoxidation of the various 2-Cys Prxs in different cellular compartments, with PrxIV being particularly resistant to exogenously added H_2_O_2_.

We have shown above that PrxIV can become hyperoxidized by an Ero1-dependent process in the presence of DTT. We evaluated whether the Ero1-generated H_2_O_2_ could also hyperoxidize PrxII or PrxIII in HT1080 cells (Fig. 4*C*). When DTT was added to cells, PrxIV was mostly hyperoxidized. However, only modest, but statistically significant (*p* <0.05), levels of hyperoxidation of PrxII and PrxIII were observed at higher concentrations of DTT. These results indicate that ER-generated H_2_O_2_ can enter the cytosol at a sufficient concentration to hyperoxidize PrxII. In addition, it is clear that, in the presence of Ero1-generated H_2_O_2_ and a reducing agent, PrxIV does become sensitive to hyperoxidation.

The differential sensitivity of the 2-Cys Prxs to hyperoxidation by exogenously added H_2_O_2_ may be explained if the individual Prxs differed in their intrinsic sensitivity to hyperoxidation. It has been demonstrated that the efficiency of reduction of the disulfide formed between the peroxidatic and the resolving cysteine of the 2-Cys Prxs has a dramatic effect on the sensitivity to hyperoxidation (6, 7), so variations in the reductive pathways in each of the cellular compartments could also account for the variations in sensitivity to hyperoxidation. Alternatively, the lack of hyperoxidation of PrxII or PrxIII by ER-generated H_2_O_2_ and the resistance of PrxIV to exogenously generated oxidant could be due to the ER membrane being impermeable to H_2_O_2_.

**FIGURE 5.**
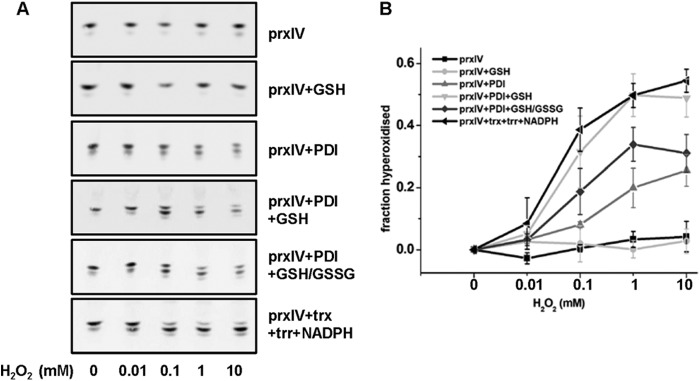
**Hyperoxidation of purified PrxIV in the presence of different reductants.** Purified human PrxIV (2.8 μm) was incubated in the presence of different reductants and in the presence of increasing concentrations of H_2_O_2_ (0–10 mm) for 10 min. Samples were then separated by one-dimensional IEF. Immunoblot analyses were developed using the rabbit polyclonal antibody to PrxIV (α-*prxIV*). The oxidized forms of PrxIV were quantified, and the fraction hyperoxidized at each concentration was calculated after subtracting the fraction hyperoxidized in the absence of added H_2_O_2_. *Error bars* represent the mean ± S.D. from three independent experiments. *trx*, thioredoxin (2.8 μm); *trr*, thioredoxin reductase (2.8 μm).

To assess whether PrxIV is intrinsically insensitive to hyperoxidation, we used purified components in an attempt to reconstitute a pathway of hyperoxidation. Note that purified recombinant PrxIV is primarily oxidized (9), so the disulfide would need to be reduced to allow oxidation. In the absence of any other components, PrxIV does not become hyperoxidized, even at 10 mm H_2_O_2_ (Fig. 5). However, in the presence of thioredoxin, thioredoxin reductase, and NADPH, PrxIV is readily hyperoxidized at concentrations of H_2_O_2_ as low as 100 μm. These results demonstrate that PrxIV will become hyperoxidized at similar concentration of H_2_O_2_ that modified PrxII in cells if a robust recycling system is present. Within the ER, the initial reductase for PrxIV is likely to be a member of the PDI family (9). Indeed, when a stoichiometric amount of reduced PDI was incubated with PrxIV, hyperoxidation occurred. The sensitivity of PrxIV to hyperoxidation was maximized when GSH (1 mm) was included with reduced PDI, conditions that, as we have shown previously, fully reduce PrxIV (10). The ER lumen is unlikely to contain such reducing conditions, so the level of hyperoxidation of PrxIV in cells will most likely reflect the prevailing ratio of GSH:GSSG. Indeed, in the presence of a 3:1 ratio of GSH:GSSG and reduced PDI, PrxIV was less sensitive to hyperoxidation than with GSH alone (*p* < 0.03). An absolute requirement for PDI in this system was evidenced by the lack of PrxIV hyperoxidation when incubated in the presence of increasing concentrations of H_2_O_2_ and GSH at 1 mm. Analysis of the extent of hyperoxidation of PrxIV confirmed that, at 10 mm H_2_O_2_, statistically significant differences were seen between the presence and absence of all reductant apart from GSH alone (*p* < 0.05 (PDI) to *p* < 0.005 (thioredoxin, thioredoxin reductase, and NADPH). Hence, the level of hyperoxidation of PrxIV reflects the efficiency of the recycling system and requires a member of the PDI family to catalyze the initial reduction of the active site disulfide.

##### Cellular PrxIV Becomes Hyperoxidized in the Presence of a Reducing Pathway

**FIGURE 6.**
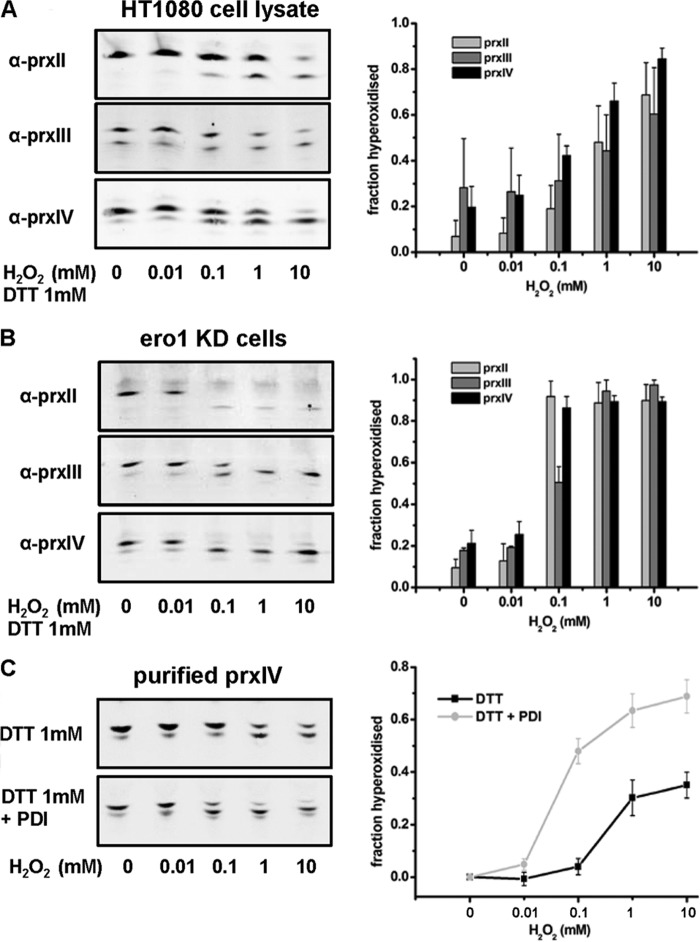
**Hyperoxidation of ER-localized PrxIV in the presence of a reducing pathway.** *A*, a HT1080 cell lysate was incubated for 10 min in the presence of DTT (1 mm) and increasing concentrations of H_2_O_2_, as indicated. Samples were then treated with *N*-ethylmaleimide and separated by one-dimensional IEF. Prxs were detected as described in Fig. 3. Data presented are representative of three independent experiments. *B*, an Ero1-depleted cell line was incubated in the presence of DTT (1 mm) and increasing concentrations of H_2_O_2_ for 10 min. Cells were treated with *N*-ethylmaleimide and lysed as described in Fig. 2 before proteins were separated by one-dimensional IEF. The experiment was carried out in triplicate with similar results. *C*, purified PrxIV (2.8 μm) was incubated with DTT (1 mm) with or without purified PDI (2.8 μm). The assays were performed and quantified as described in Fig. 5.

The results with purified protein components provide compelling evidence that PrxIV can become hyperoxidized at quite low concentrations of H_2_O_2_. To determine whether an equivalent sensitivity of the three Prxs to hyperoxidation could occur when they were exposed to the same recycling pathway, we first lysed cells with detergent and then assessed the sensitivity of the three Prxs to increasing concentrations of H_2_O_2_ (Fig. 6*A*). Under these conditions, the cytosolic and mitochondrial reduction pathways would be diluted, so to provide a source of reducing equivalents, we included DTT (1 mm). Each of the Prxs was now sensitive to hyperoxidation, with PrxIV as sensitive as PrxII.

To determine whether PrxIV would be as sensitive as the other Prxs when a source of reductant was present in the ER, we took advantage of the fact that cells depleted of Ero1α do not produce H_2_O_2_ when DTT is added (Fig. 2*B*). Hence, we can add DTT to these cells, where it will act as a reductant, directly or indirectly, for PrxIV without generating H_2_O_2_. When the Ero1α-depleted cells were incubated with H_2_O_2_ in the presence of DTT, PrxIV became as sensitive to hyperoxidation as PrxII (>80% hyperoxidation at 100 μm H_2_O_2_) (Fig. 6*B*). This result demonstrates that ER-localized PrxIV is sensitive to hyperoxidation when a reducing agent is included and explains why PrxIV is sensitive to hyperoxidation in non-Ero1α-depleted cells when DTT is present. In addition, it shows that H_2_O_2_ must be able to permeate the ER membrane, proving that the lack of hyperoxidation of PrxIV was not due to a lack of H_2_O_2_ in the ER.

The DTT added to cells could reduce PrxIV directly or indirectly via PDI. To determine whether DTT can directly accelerate the recycling of PrxIV and lead to hyperoxidation, we incubated purified PrxIV with DTT and added increasing concentrations of H_2_O_2_ (Fig. 6*C*). The presence of DTT sensitized PrxIV to hyperoxidation. However, this effect was more pronounced when PDI was also included in the incubation. Taken together with the more efficient hyperoxidation of PrxIV in the presence of PDI and GSH (Fig. 5), we conclude that PDI or a PDI family member is the primary reductant of PrxIV and that maximal sensitivity can be achieved when either DTT or GSH is present to reduce PDI. In the lumen of the ER, PrxIV is sensitive to hyperoxidation only when PDI is efficiently reduced with DTT. The resistance of PrxIV to hyperoxidation indicates that an efficient mechanism to reduce PDI is absent from the ER.

## DISCUSSION

We developed a simple quantitative assay to address the extent of hyperoxidation of members of the 2-Cys family of Prxs. This assay, on the basis of separation by one-dimensional IEF of the hyperoxidized from the unmodified form, can be used to evaluate the efficiency of the thiol reduction pathways in different intracellular organelles. The assay could also be used to quantify hyperoxidation of Prxs during the cell cycle (20) or to determine whether the oxidation status of Prxs changes during circadian oscillations (21). Here we used the assay to assess the sensitivity of Prxs in different cellular compartments to hyperoxidation following exposure to H_2_O_2_. Although the pathway for reduction of PrxII and PrxIII is fairly well characterized, the pathway for reduction of PrxIV was unclear. It has been shown previously that the disulfide formed in PrxI, II, and III following sulfenylation of the peroxidatic cysteine is reduced by a thioredoxin, which itself is reduced by a thioredoxin reductase present in the cytosol (PrxI and II) or mitochondria (PrxIII), with the ultimate electron donor being NADPH (6, 7). The fact that both Prx II and III were sensitive to hyperoxidation in the presence of H_2_O_2_ confirms that the thioredoxin reduction pathway present in the cytosol or the mitochondria was not compromised in the presence of this oxidant. The absence of hyperoxidation of PrxIV suggests that no equivalent pathway exists in the ER lumen for the reduction of thioredoxin domain-containing proteins. The presence within the ER of several thioredoxin domain-containing proteins that make up the PDI family allows the initial reduction of PrxIV (10). In the absence of a thioredoxin reductase to reduce the PDI family member, the disulfide within PrxIV will persist, thereby protecting the peroxidatic cysteine from hyperoxidation.

The protection of Prx from hyperoxidation means that, under normal physiological conditions, it is unlikely that PrxIV becomes extensively inactivated by this mechanism. This is in contrast to the other Prxs, which are regulated by hyperoxidation to allow H_2_O_2_ to act as a signaling molecule and modify proteins such as members of the protein tyrosine kinase family (22). In contrast to the cytosolic enzymes that are recycled from their hyperoxidized form by sulfiredoxin, hyperoxidized PrxIV could not be recycled, suggesting that there is no ER-localized sulfiredoxin.

Many studies regarding disulfide formation in the ER have used DTT as an experimental tool to prevent disulfide formation during a short pulse and, upon its removal, have followed the posttranslational formation of disulfides (23, 24). The assumption has been that the effect of DTT over a short pulse was to prevent disulfide formation because the reagent has no effect on the secretion of non-disulfide-bonded proteins. The demonstration that DTT also generates H_2_O_2_ in an Ero1-dependent fashion and that this leads to the hyperoxidation and inactivation of PrxIV should be taken into account when interpreting these previous studies. It should be noted that other peroxidases, such as Gpx7 or 8, could contribute to the removal of H_2_O_2_ under these conditions (25). However, like PrxIV, both these enzymes require a disulfide exchange protein to maintain optimal activity. Although it is clear that the ER redox status rapidly recovers from the added DTT by an Ero1-dependent process (17, 26), the inactivation of PrxIV will have a consequence for the metabolism of H_2_O_2_ for several hours following the addition of DTT. Hence, the interpretation of the effect of DTT addition to cells on their ability to recover ER redox status, to form disulfides, and to mount a stress response needs to take into account the fact that H_2_O_2_ metabolism will be impaired because of the inactivation of PrxIV.

Studies into the variation in the sensitivity of the 2-Cys peroxiredoxin family members to hyperoxidation has focused on differences between the intrinsic ability of each enzyme to be inactivated by H_2_O_2_ (5). Using purified protein, it has been shown that PrxIII is more resistant to hyperoxidation than PrxII because of subtle sequence differences within the C-terminal region. In contrast to PrxIII, we show here that PrxIV is resistant to hyperoxidation because of a lack of an efficient recycling system in the ER. To demonstrate that the lack of hyperoxidation was not due to an intrinsic resistance of this enzyme, we showed that purified Prx IV was susceptible to hyperoxidation in the presence of either a thioredoxin recycling system or PDI with a reducing agent such as DTT or GSH. In addition, ER-localized PrxIV also became sensitive to hyperoxidation in the presence of DTT. These results confirm the requirement for a PDI family member to directly reduce PrxIV in the ER (10, 27) and show that the oxidized PDI can be efficiently reduced in the presence of a reducing GSH buffer or DTT. The requirement for a GSH buffer to maintain a reductive pathway in the ER has been suggested previously because lowering the levels of intracellular GSH does not prevent disulfide formation but does lead to the persistence of non-native disulfides in the secreted protein tissue-plasminogen activator (28). The resistance of PrxIV to hyperoxidation in the ER indicates that an efficient pathway for the reduction of GSSG is also absent from the ER. Hence, a mechanism for GSSG/GSH exchange between the ER and the cytosol most likely exists to maintain a sufficiently reducing GSH:GSSG ratio to facilitate the reduction of members of the PDI family and allow non-native disulfide reduction.

## Footnotes

2 The abbreviations used are:

Prx: peroxiredoxin
ER: endoplasmic reticulum
PDI: protein disulfide isomerase
IEF: isoelectric focusing
CMVLS: College of Medical, Veterinary and Life Science.
